# Dr. Henry H. Langworthy

**Published:** 1889-04

**Authors:** 


### Obituary.

Dr. John W. Southworth, of Rochester, died March 17th, at the age of 46 years. During the war, he served as hospital steward, and after its close began the study of medicine, and was graduated from the University of Michigan in 1867. The early years of his professional life were spent in several places, but for the past twelve years he has practiced in Rochester. He was a member of the County Medical Society, at a meeting of which appropriate resolutions of respect and sympathy were passed.

Dr. Henry H. Langworthy died at Rochester February 5, 1889, aged 61 years. He was graduated from the Geneva Medical College in 1848, and shortly afterward settled in Rochester, where he has been engaged in the active practice of his profession until about three years ago, when his health began to fail. At a special meeting of the Monroe County Medical Society, February 6th, a number of his professional brethren paid high tribute to his character and worth. The following resolution was adopted as an expression of the esteem in which the deceased was held:

" *Whereas*, Death having removed from our midst our esteemed colleague, Dr. H. H. Langworthy, we, in society assembled, desire to testify to his great worth, both as a man and a physician. During a long life in the practice of an arduous profession, no stain rests on his memory. His mind was constantly occupied with matters of an elevated character. We therefore resolve that these characteristics should be placed upon record, and be published in the daily papers."

At a subsequent meeting of the Rochester Pathological Society, of which Dr. Langworthy was an honorary member, similar tribute was paid to his memory. In Dr. Langworthy's death, Rochester loses one of her oldest and most accomplished physicians.

